# Spontaneous Angiolipoma in Autologous Flap Reconstruction

**DOI:** 10.7759/cureus.31582

**Published:** 2022-11-16

**Authors:** Puja Shahrouki, Tiffany L Chan, Hyung Won Choi, Anthony H Chau, Lucy Chow

**Affiliations:** 1 Radiology, University of California, Los Angeles, Los Angeles, USA; 2 Radiology, University of California, Irvine, Irvine, USA; 3 Vascular Surgery, University of California, Irvine, Irvine, USA

**Keywords:** autologous flap reconstruction, ultrasound, mammography, magnetic resonance imaging, breast cancer recurrence, angiolipoma

## Abstract

Breast cancer recurrence after autologous flap reconstruction is rare and typically occurs at the contact zone between the flap and the native tissue. When a new lesion is found in a reconstructed breast without the characteristic appearance of benign entities such as fat necrosis, definitive tissue diagnosis is often warranted to rule out recurrence or metastasis. Angiolipomas are rare, benign lipomatous tumors that have nonspecific imaging appearances and are thus frequently biopsied or excised for definitive diagnosis. Here, we report a case of a new breast mass found at the contact zone of a reconstructed breast in a patient with a history of ductal carcinoma in situ (DCIS), which was ultimately excised and proven to be an angiolipoma.

## Introduction

Breast cancer recurrence after mastectomy is rare, regardless of whether skin-sparing mastectomy, nipple-sparing mastectomy, or modified radical mastectomy is performed [1]. Although the theoretical risk of recurrence is virtually eliminated in the case of ductal carcinoma in situ (DCIS) [2], metachronous breast cancer may occur in the remaining native tissue, especially in patients with genetic risk factors [3]. Although the risk of recurrence is not altered by whether or not breast construction is performed after mastectomy, benign postsurgical changes associated with reconstruction may present additional diagnostic challenges when abnormalities are found in the reconstructed breast [3].

Here, we report a case of a new mass arising at the junction of the residual breast and autologous flap in a patient with a history of breast cancer, proven to be an angiolipoma.

## Case presentation

**Figure 1:**
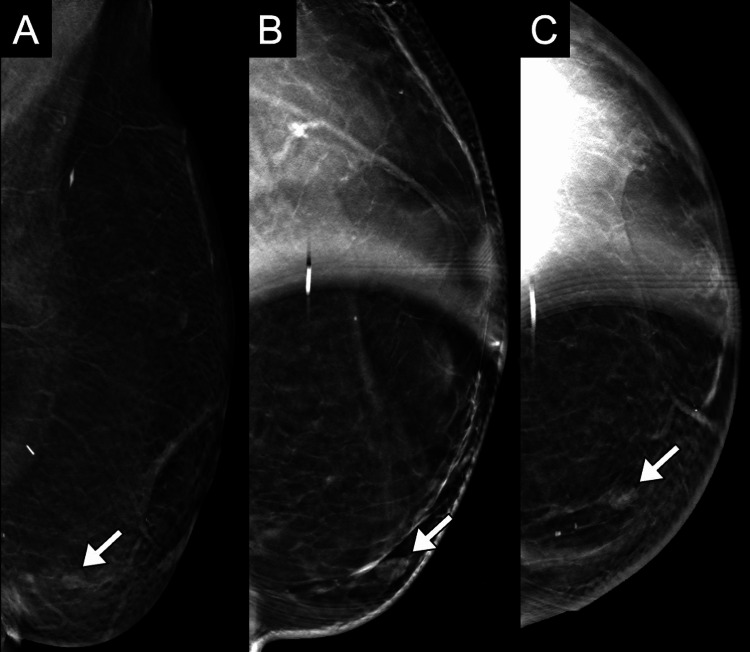
Diagnostic digital tomosynthesis of the left breast. (A) ML, (B) spot MLO, and (C) spot CC views confirm the presence of an 8-mm circumscribed, oval, equal-density mass (arrows) in the lower inner quadrant, 7 cm from the nipple, at the contact zone between the flap and the native tissue. ML: mediolateral, MLO: mediolateral oblique, CC: craniocaudal

**Figure 2:**
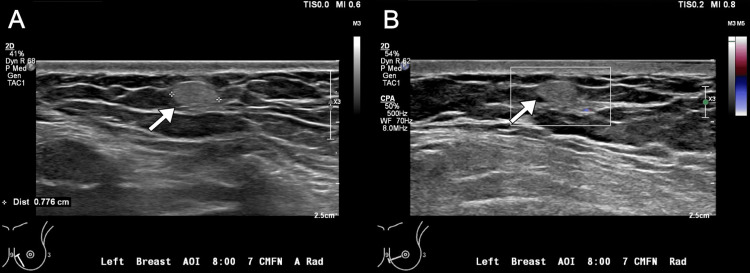
Diagnostic sonographic images of the left breast. (A) Grayscale and (B) color Doppler images demonstrate an 8-mm circumscribed, oval, homogeneously hyperechoic mass (arrows) without associated vascularity superficially in the lower inner quadrant of the breast, 7 cm from the nipple.

A 64-year-old female with a history of left breast DCIS status post mastectomy with muscle-sparing free transverse rectus abdominis muscle flap reconstruction without evidence of recurrence for the past 10 years presented for routine screening mammography. A new mass was found in the lower inner quadrant of the left breast, located 3 cm from the nipple, on the screening mammogram. The patient was recalled for further evaluation (Breast Imaging Reporting and Data System (BI-RADS) Category 0) [4], whereupon diagnostic mammogram (Figure 1) and ultrasound (Figure 2) showed an oval, circumscribed, homogeneously hyperechoic mass in the left breast.

**Figure 3:**
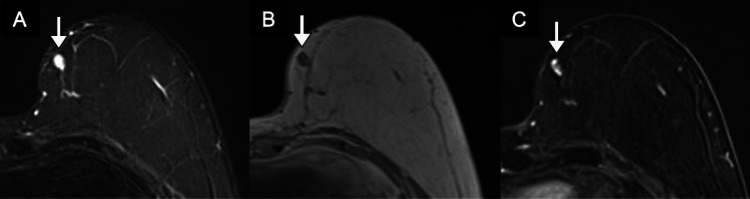
Surveillance magnetic resonance imaging of the left breast. (A) Axial STIR, (B) pre-contrast T1, and (C) post-contrast T1 fat-saturated images show an 8-mm oval, enhancing STIR hyperintense mass (arrows) located superficially at the contact zone. The mass demonstrated slow progressive enhancement (type 1 curve). A few additional small enhancing foci are seen along the breast-flap interface. STIR: short tau inversion recovery

Given the homogeneously hyperechoic appearance on ultrasound possibly representing a benign entity such as fat necrosis, a BI-RADS Category 3 was assigned. Subsequently, a month later, the patient underwent a routine surveillance breast magnetic resonance imaging (MRI) examination, which demonstrated that the previously seen mass in the left breast did not have any fat signal (Figure 3).

**Figure 4:**
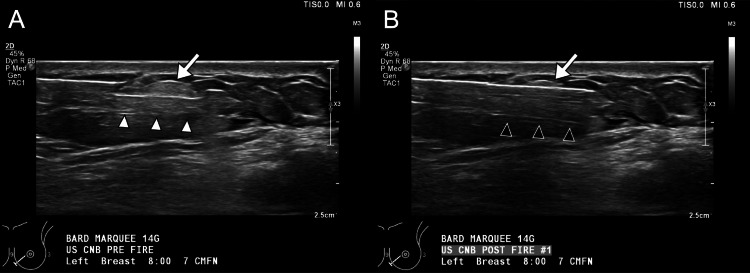
Ultrasound-guided core needle biopsy of the superficial mass in the left breast at the contact zone. (A) Pre-fire and (B) post-fire grayscale images show successful placement (white arrowheads) and deployment (black arrowheads) of core needle biopsy of the mass (arrow).

In the absence of an associated fat signal, the mass was now assigned a BI-RADS Category 4 with a recommendation for an ultrasound-guided core needle biopsy for definitive diagnosis (Figure 4).

**Figure 5:**
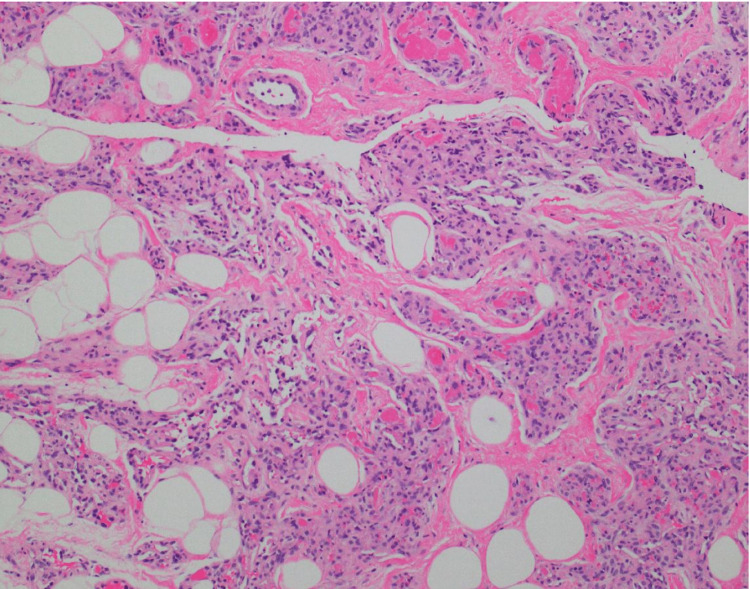
H&E stain from the biopsy of the superficial mass in the left breast. Low-grade vascular neoplasm in a background of fibroadipose tissue. H&E: hematoxylin and eosin

Histopathology demonstrated a low-grade vascular neoplasm with features suggestive of an angiolipoma (Figure 5). Surgical excision was pursued to rule out a higher-grade lesion with the final histopathology showing an angiolipoma without atypical hyperplasia or carcinoma.

## Discussion

Angiolipomas in the breast are uncommon tumors containing mature fat cells and vascular proliferation [5]. In the few cases reported so far in the literature, patients typically present with a painless mass or a nonpalpable, incidental mass found on imaging [6-8]. Angiolipomas occurring in other parts of the body, like the trunk and extremities, typically present as painful masses [5,8]. Angiolipomas are typically less than 2 cm in size and may be solitary or multiple in number [5-7]. Their imaging appearance on mammography, ultrasound, and MRI is nonspecific [5-7,9], although the most characteristic finding is a homogeneously or heterogeneously hyperechoic mass, with associated vascularity and no posterior acoustic shadowing [6,7]. Main differential considerations include other benign entities that may present as hyperechoic masses, commonly fat necrosis and hematoma, or, in rare cases, spindle cell lipoma, another rare benign fat-containing tumor [3,5,10]. Hemangiomas, one of the more common entities, can also be hyperechoic on ultrasound but tends to demonstrate posterior acoustic shadowing and may rarely, although characteristically, have associated calcifications (phleboliths) [5,6]. Although a breast hematoma may also be hyperechoic, it tends to be more heterogeneous in echogenicity and often has an associated history of trauma or surgery [5]. Lipomas of the breast have echogenicity similar to the fat tissue that is hypoechoic compared to the relatively hyperechoic fibroglandular tissue of the breast [5,9]. In addition, lipomas are typically avascular [9]. When a mass demonstrates irregular margins, suspicious calcifications, thick hyperechoic capsule, or increasing size, malignancy should be considered [5,8]. Heterogeneously hyperechoic angiolipomas may be indistinguishable from some breast cancers such as invasive carcinoma, liposarcoma, and angiosarcoma [11]. Mucinous carcinomas may present as a particular diagnostic dilemma as they are T2 hyperintense on MRI and can in rare cases present as a heterogeneously hyperechoic mass on ultrasound [12,13]. However, if a homogeneously hyperechoic mass is seen on ultrasound without posterior acoustic shadowing and there are no other suspicious findings on additional imaging, both angiolipoma and spindle cell lipomas should be entertained as likely differential considerations. Neither has known malignant potential, but because of the paucity of knowledge about these entities, these are frequently biopsied and surgically excised [6,9,10]. This may be especially true when there is a history of breast cancer, as was the case with our patient (Table 1).

**Table 1:** Angiolipoma of the breast. *The most typical imaging findings are provided, although other findings have been described in isolated cases. **The most specific imaging appearance. US: ultrasound, MRI: magnetic resonance imaging

Angiolipoma of the breast	
Clinical findings	Painless, palpable mass
	Painless, nonpalpable mass
Imaging findings*	Mammogram: round or oval, iso- or hypodense <2 cm mass with circumscribed margins
	US: Circumscribed, homogeneously hyperechoic** or heterogeneously hyperechoic <2 cm mass with increased vascularity and no posterior acoustic shadowing
	MRI: T1 hypointense, T2 hyperintense <2 cm mass with enhancement
Differential diagnoses	Benign: spindle cell lipoma, hematoma, lymph node, lipoma
	Malignant: invasive carcinoma, liposarcoma, angiosarcoma

Breast cancer recurrence after mastectomy is rare, especially in DCIS [2]; however, in patients with genetic risk factors, such as breast cancer 1 (*BRCA1*) and breast cancer 2 (*BRCA2*), the risk of recurrence is increased [3]. There is no difference in recurrence rates between skin-sparing mastectomy, nipple-sparing mastectomy, or modified radical mastectomy [1]. While breast reconstruction is not known to change the risk of recurrence, attention to typical recurrent breast cancer locations on imaging is important. When recurrence occurs in patients with autologous flap reconstruction, it is typically found superficially at the medial aspect of the flap where there is more likely to be residual breast tissue or posteriorly near the chest wall, deep to the muscular layer of the flap [3,14]. There have been case reports describing recurrence in the flap, without surrounding breast tissue on histopathology, which may be owed to direct spread from residual tumor cells, tumor seeding during surgery, or metastatic foci already present within the flap before surgery [15]. Benign postsurgical changes associated with autologous flaps, such as hematoma and fat necrosis, may present an additional diagnostic challenge, although these typically develop immediately postoperatively to weeks after the surgery, as opposed to cancer recurrence, which tends to present years later [3]. Fibrosis may develop up to 18 months postoperatively and may present as an irregular mass with enhancement, which may be difficult to differentiate from recurrence [3].

## Conclusions

This case highlights the importance of awareness of breast cancer recurrence patterns in autologous flap reconstructions and rare benign breast masses such as angiolipoma and spindle cell lipoma. A homogeneously hyperechoic mass with vascularity and no posterior acoustic enhancement is suggestive of a benign entity such as an angiolipoma in this case; however, if nonspecific imaging findings are seen or if the patient has a history of breast cancer, definitive diagnosis with tissue sampling and/or surgical excision may be indicated to exclude malignancy.
